# R2R3-MYB transcription factors, StmiR858 and sucrose mediate potato flavonol biosynthesis

**DOI:** 10.1038/s41438-021-00463-9

**Published:** 2021-02-01

**Authors:** Sen Lin, Rajesh K. Singh, Moehninsi, Duroy A. Navarre

**Affiliations:** 1 Irrigated Agriculture Research and Extension Center, Washington State University, Prosser, WA USA; 2 USDA-Agricultural Research Service, Prosser, WA USA; 3 Present Address: Department of Biotechnology, CSIR-Institute of Himalayan Bioresource Technology, Palampur, Himachal Pradesh 176061 India

**Keywords:** Secondary metabolism, Plant molecular biology

## Abstract

Flavonols and other phenylpropanoids protect plants from biotic and abiotic stress and are dietarily desirable because of their health-promoting properties. The ability to develop new potatoes (*Solanum tuberosum*) with optimal types and amounts of phenylpropanoids is limited by lack of knowledge about the regulatory mechanisms. Exogenous sucrose increased flavonols, whereas overexpression of the MYB StAN1 induced sucrolytic gene expression. Heterologous StAN1 protein bound promoter fragments from sucrolytic genes (*SUSY1* and *INV1*). Two additional MYBs and one microRNA were identified that regulated potato flavonols. Overexpression analysis showed *MYB12A* and *C* increased amounts of flavonols and other phenylpropanoids. Endogenous flavonol amounts in light-exposed organs were much higher those in the dark. Expression levels of *StMYB12A* and *C* were high in flowers but low in tubers. Transient overexpression of miR858 altered potato flavonol metabolism. Endogenous StmiR858 expression was much lower in flowers than leaves and correlated with flavonol amounts in these organs. Collectively, these findings support the hypothesis that sucrose, MYBs, and miRNA control potato phenylpropanoid metabolism in a finely tuned manner that includes a feedback loop between sucrose and StAN1. These findings will aid in the development of potatoes with phenylpropanoid profiles optimized for crop performance and human health.

## Introduction

Potato (*Solanum tuberosum*) is one of the world’s staple foods, along with rice, wheat and corn, and will be instrumental in achieving global food security, given projections that crop yields need to double by 2050^1^. One aspect of food security is the ability to meet energetic and nutritional needs. In addition to providing energy from complex carbohydrates, potatoes contain various vitamins, minerals and phytonutrients, including Vitamins B_6_, B_9_, and C, potassium and carotenoids^2–8^. Potatoes also contain phenylpropanoids, which are dietarily desirable because of their beneficial effects on health thought to include cardiovascular health, longevity, gut and eye health, along with anti-inflammatory and chemoprotective properties^9–12^.

Phenylpropanoids accumulate constitutively and may also be induced by environmental conditions. They have diverse roles in plants such as growth and development, and in modulating plant interactions with environment, including microbes, insects, and abiotic stress^13–16^. Potatoes, especially those with purple, blue, or red-flesh can contain high amounts of phenylpropanoids such as chlorogenic acids and anthocyanins, but low amounts of flavonols, which can be induced by wounding^17–22^.

**Fig. 1:**
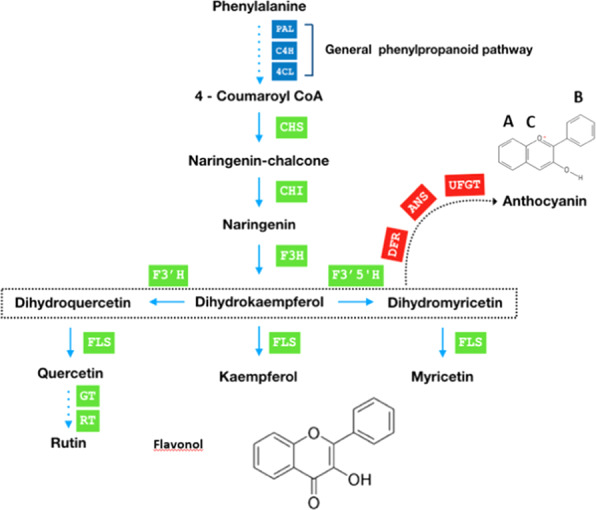
Simplified schematic representation of flavonol biosynthesis pathway. PAL phenylalanine ammonia lyase, C4H cinnamate 4-hydroxylase, 4CL 4-coumaroyl:CoA-ligase, CHS chalcone synthase, CHI chalcone isomerase, F3H flavanone 3-hydroxylase, F3′H flavonoid 3′ hydroxylase, F3′5′H flavonoid 3′,5-hydroxylase, FLS flavonol synthase, DFR dihydroflavonol 4-reductase, ANS anthocyanin synthase

With over 8000 metabolites, flavonoids are the largest class of polyphenols and about 20% of the total carbon flux in a plant cell goes through the flavonoid pathway^23^. Flavonoids such as anthocyanins and flavonols are synthesized from p-coumaroyl-CoA and three malonyl-CoA molecules by chalcone synthase, a type III polyketide synthase, in the first committed step (Fig. 1a). Chalcone isomerase (CHI) catalyzes the intramolecular cyclization of chalcones to (2S)-flavanones, which are a common substrate for multiple branches of the flavonoid pathway. Ectopic expression of CHI in tomato increased flavonol amounts over 70-fold^24,25^. Flavanone 3-hydroxylase (F3H) converts flavanones into dihydroflavonols, which are precursors for both anthocyanins and flavonols^26^. Flavonols have a C3 hydroxyl group and a C2–C3 double bond, with the committed step in the pathway catalyzed by flavonol synthase (FLS), a member of the 2-oxoglutarate-dependent dioxygenase (2-ODD) superfamily^27^. The first committed step in the anthocyanin pathway is catalyzed by dihydroflavonol reductase (DFR), which competes with FLS for dihydroflavonol precursors. FLS expression is regulated by transcription factors, including MYB12^28^.

MYBs regulate numerous processes in plants, including reproduction, cell division, responses to biotic and abiotic stresses, and secondary metabolism^29–32^. In silico analysis were identified over 230 candidate MYB genes in potato, several of which were induced by hormone treatments, salt or drought stress^33,34^. Overexpression of MYB12 in tomato increased the flavonol content^35^. Expression of *CHS*, *CHI*, and *FLS* was upregulated in Arabidopsis by overexpressing *MYB12*, but drastically decreased in a *myb12* mutant^28^. Both chlorogenic acid and anthocyanin synthesis in potato are regulated by the R2R3 MYB transcription factor stAN1, but little is known about the endogenous MYB12 in potato.

In addition to MYBs, small RNAs can regulate phenylpropanoid metabolism^15^. MicroRNAs control gene expression at the transcriptional and post-transcriptional levels, and regulate many biological processes including secondary metabolism^36–41^. Increasing miR857 levels in Arabidopsis decreased the lignin content^42^, while overexpression of miR408 specifically downregulated phenylalanine ammonia lyase (*PAL*) genes in transgenic *Populus*^43^. MiR828 regulates plant phenylpropanoid accumulation by targeting R2R3 MYBs, and is associated with anthocyanin expression in potato^15,44^. Expression of miR858 is associated with flavonol metabolism in various plants, and also with susceptibility to cyst nematodes^45–49^.

Potatoes with higher amounts of flavonols are dietarily desirable. However, while potatoes high in phenylpropanoids such as anthocyanins and chlorogenic acid are known, no high-flavonol potato has been identified. The reason for this is not clear, and the flavonol branch of the potato phenylpropanoid pathway has been less studied than the anthocyanin or hydroxycinnamic acid branches. A lack of knowledge about how tuber phenylpropanoid metabolism is regulated, impedes the ability to develop new cultivars with the optimized types and amounts. Previously, a functional analysis of the MYB transcription factor StAN1 (Anthocyanin 1) showed its effect were not specific to anthocyanins, but that hydroxycinnamic acids and flavonols were also affected. We hypothesized a self-regulatory loop may exist between sucrose and StAN1^50^. In the current study, we present functional evidence that StAN1 interacts with the promoters of sucrolytic genes, and identified three additional regulators of potato flavonol metabolism, StMYB12A, C, and miR858.

## Results

### StAN1 and sucrose in potato phenylpropanoid metabolism

**Fig. 2:**
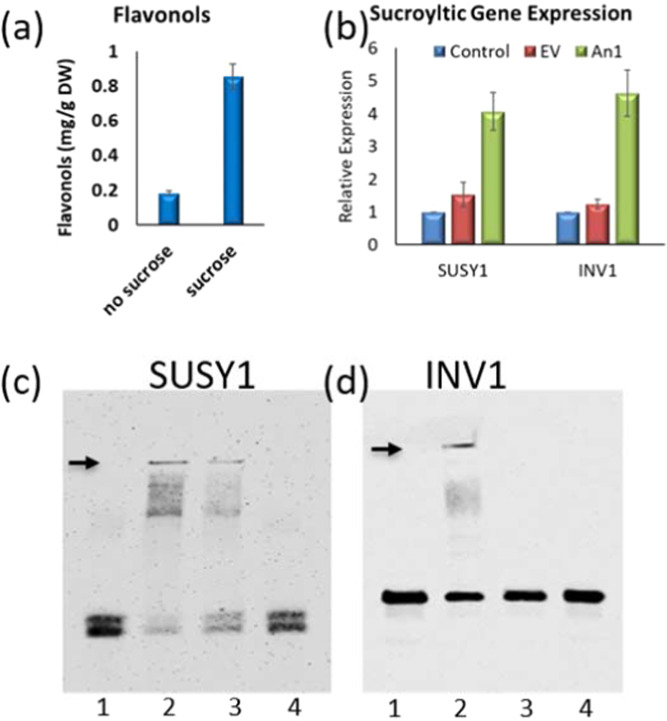
Sucrose, MYB, and flavonol interactions. **a** Flavonol content in potato plantlets grown in tissue culture with or without 120 mM sucrose. **b** Effect of AN1 infiltration on expression of genes involved in sucrose metabolism. **c** EMSA gels. All lanes have biotin labeled SUSY1 promoter fragment. Lane 1, minus StAN1 (control); Lane 2, StAn1 purified protein; Lane 3, StAN1 protein plus a 5-fold excess of cold competitor; Lane 4, StAN1 with a 50× excess of cold competitor. **d** EMSA gels. All lanes have biotin labeled INV1 promoter fragment. Lane 1, minus StAN1 (control); Lane2, StAn1 purified protein; Lane 3, StAN1 protein plus a 5-fold excess of cold competitor; Lane 4, StAN1 with a 50× excess of cold competitor

Previous work showed StAN1 overexpression increased not only anthocyanins, but other phenylpropanoids, including flavonols. The *StAN1* promoter was found to have multiple sucrose responsive elements (SURE) and sucrose feeding induced phenylpropanoids^50^. We hypothesized that StAN1 overexpression affected multiple phenylpropanoid branches due, in part, to its effect on sucrose. Growing potato plantlets in tissue culture in the presence of 120 mM sucrose increased the amount of flavonols present, and infiltration of potato leaves with *StAN1* increased expression of genes involved in sucrose breakdown (Fig. 2). The promoter regions of potato *SUSY1*, *SUSY4*, *INV1*, and *INV2* genes have MYB binding domains (Fig. S1), suggesting that StAN1 may interact directly with the promoters of sucrolytic genes. To examine whether StAN1 directly interacts with sucrolytic genes, electrophoretic mobility shift assays were conducted using heterologous StAN1 protein purified from *E. coli* (Fig. S2) and promoter fragments from *SUSY1* and *INV1*. StAN1 interacted with the promoters of both genes (Fig. 2c, d). Similar results were seen for *SUSY4* and *INV2* (data not shown).

### MYB12 A, B, and C in potato tubers

**Fig. 3:**
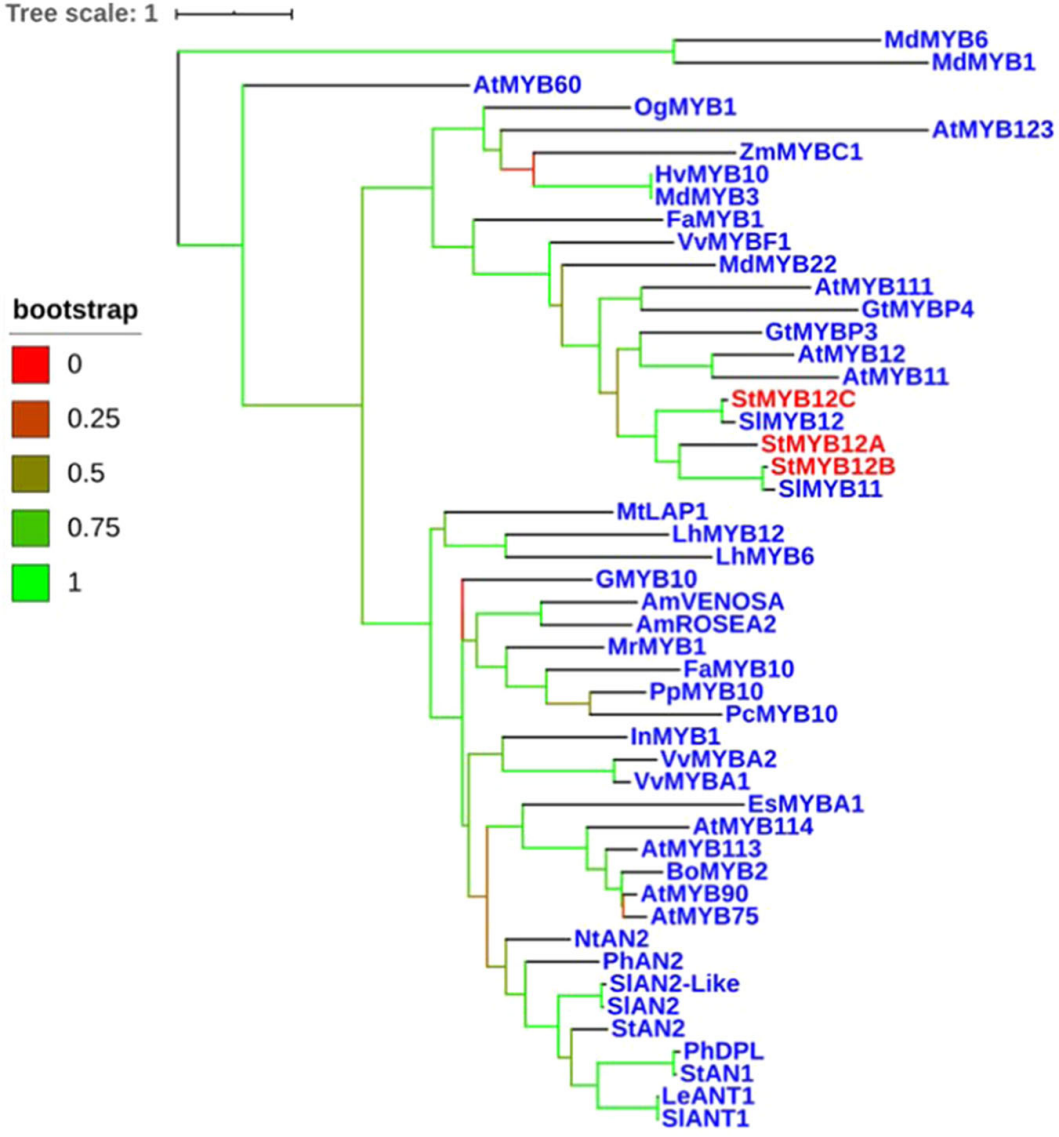
Phylogenetic tree for StAN1, StMYB12A, B, and C with other plant homologs. At *Arabidopsis thaliana*, Gt *Gentiana trifloral*, Md *Malus domestica*, Sl *Solanum lycopersicum*, St *Solanum tuberosum*, Vv *Vitis vinifera*, Zm *Zea mays*, Og *Oncidium Gower*, Hv *Hordeum vulgare*, Fa *Fragaria ananassa*, Lh *Lilium hybrid*, Am *Antirrhinum majus*, Ph *Petunia hybrida*, Nt *Nicotiana tabacum*, Bo *Brassica oleracea*, Mr Myrica rubr, Es *Epimedium sagittatum*, Pp *Prunus persica*, Pc *Pyrus calleryana*, Le *Lycopersicum esculentum*, In *Ipomoea nil*, GMYB10 is MYB10 in Gerbera. Bootstrap value from 0 to 1 are shown in different colors

While StAN1 overexpression could be used to increase flavonols, anthocyanins are its main target and would also increase, which is not desirable in many types of commercial potatoes. Nor is it obvious how sucrose levels could be used commercially to increase flavonols. To identify other factors that regulate potato flavonol biosynthesis, a BLAST search was conducted for potato homologs of the MYB12 transcription factors known to be involved in flavonol regulation in plants like tomato and Arabidopsis. The three most homologous StMYB12 candidates were named StMYB12A, B, and C and were located on chromosomes 6, 12, and 1, respectively. Relative to the Arabidopsis flavonol transcription factors, AtMYB12, AtMYB111, and AtMYB11, StMYB12C and StMYB12A were most similar to AtMYB12 and AtMYB111, respectively (Fig. S3 and Table S1). StMYB12B had more similarity with AtMYB12 (47.6.6%) than it did AtMYB11 or AtMYB111, but its similarity with AtMYB12 was less than that shared by StMYB12A (50.6%) or C (51.9%). A phylogenetic tree was generated by aligning the potato MYB12s with MYBs involved in flavonol biosynthesis in other plant species (Fig. 3). This showed that StMYB12A and B were more closely related to each other than to StMYB12C, and that StMYB12B was most closely related to SlMYB11. StMYB12C was most related to SlMYB12. StMYB12A, B, and C were all more related to each other than to any of the AN1 MYBs. All the flavonol-related MYB transcription factors were highly conserved in the N-terminus, which contains the R2 and R3 domains (Fig. S3). The DW motif adjacent to the C-terminus was also conserved. Similar to what was observed with StAN1, analysis of the MYB12 promoters detected multiple sucrose responsive elements (SURE) in *StMYB12 A* and *C*, but not B (Table S2).

### Phenylpropanoids concentrations and *MYB12* expression in different cultivars

As the first step to determine if these candidate MYB12s were involved in potato flavonol regulation, we examined whether correlations existed between endogenous flavonol amounts and StMYB12 expression in different genotypes. StMYB12C was the most homologous to AtMYB12 and SlMYB12, so flavonol amounts and *MYB12C* expression were measured in ten potato genotypes with white flesh. *MYB12C* expression varied over 8-fold among these genotypes, and was highest in La Belle Russet, which also had the highest amounts of rutin and kaempferol (Fig. S4a, b, c). Relatively low flavonol amounts were present in all of these white-flesh potatoes. A positive correlation was observed between *MYB12C* expression and flavonol amounts (Fig. S4d, e).

**Fig. 4:**
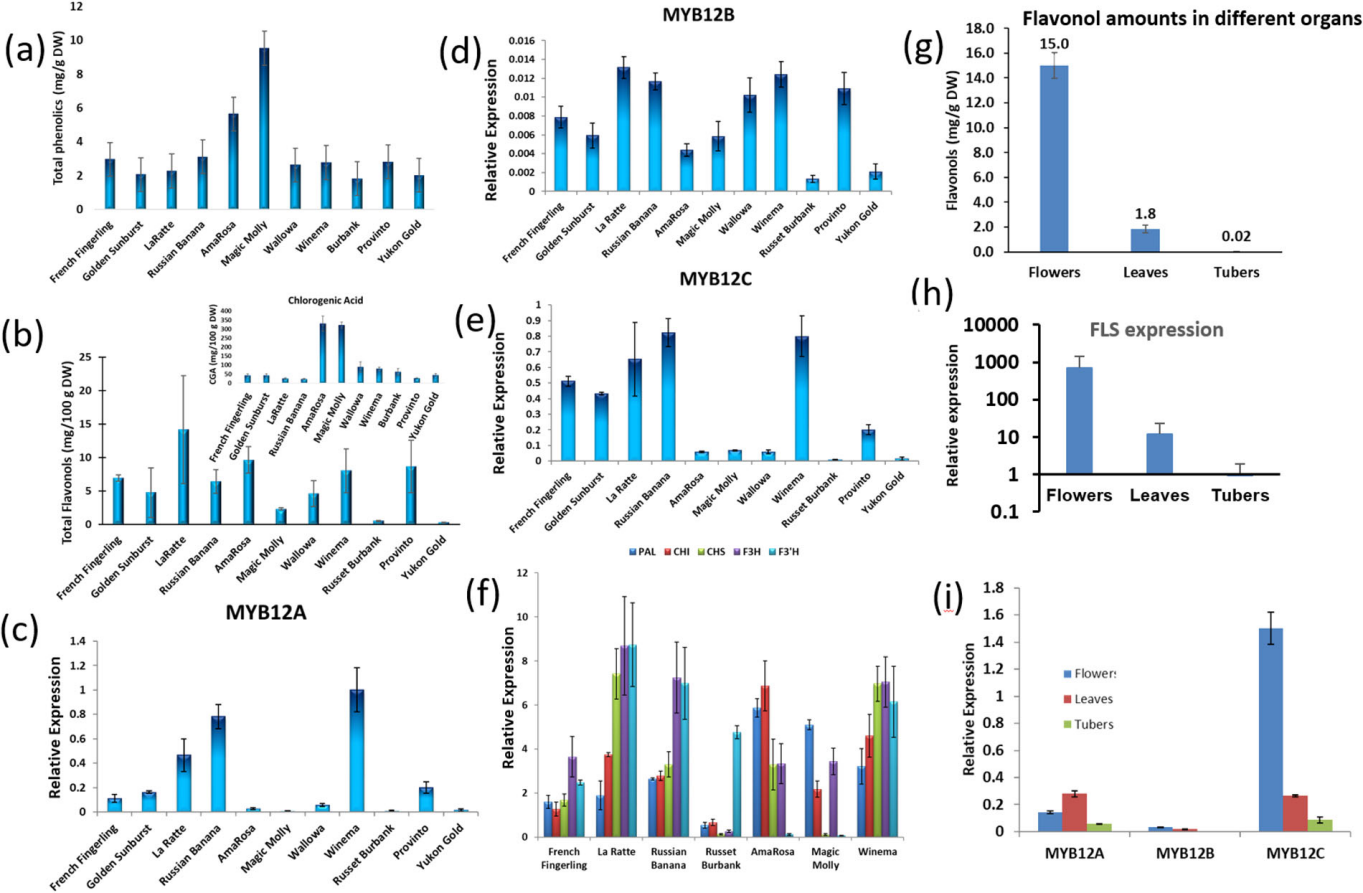
Phenylpropanoids and gene expression in different cultivars. Total phenolics (**a**) and total flavonols (**b**) in different genotypes. Chlorogenic acid amounts are shown in the inset. Relative expression of **c** MYB12A, **d** 12B, and **e** 12C in potatoes of varying flesh-color. **f** PAL and early flavonoid pathway gene expression. **g** Flavonol amounts in flowers, leaves or tubers; **h** FLS expression level in flowers, leaves and tubers; **i** Expression analysis of Myb12 A, B, and C in different organs. The data represents the means ± standard deviation of three biological replicates

Potatoes with blue-flesh, red-flesh, or purple-flesh are known to contain much higher amounts of phenylpropanoids, so we analyzed whether flavonol content and MYB expression was higher in color-flesh potatoes with more active phenylpropanoid metabolism. Flavonol content was analyzed in tubers from 11 additional cultivars representing potatoes with yellow, red, and purple flesh color in addition to white. Flavonol, chlorogenic acid, and total phenolic amounts varied markedly among these genotypes (Fig. 4), but flavonol amounts were unrelated to the amount of total phenylpropanoids in a genotype. AmaRosa and Magic Molly are cultivars with red-flesh or purple-flesh and had much higher amounts of total phenolics (Fig. 4a) and chlorogenic acid (Fig. 4b) than the white-flesh cultivars, yet Magic Molly had among the lowest amounts of flavonols (Fig. 4b).

Although StMYB12C had the highest similarity to AtMYB12 and its expression correlated with flavonol amounts in white potatoes (Fig. S4d, e), StMYB12A and B also had similarity to AtMYB12, so expression of all three transcription factors was examined. *MYB12A* and *C* were more highly expressed than *MYB12B* in all cultivars (Fig. 4c–e). Generally, potatoes with higher level of *MYB12A* and *C* expression tended to have higher amounts of flavonols, although there were exceptions such as in AmaRosa.

Expression of *PAL* and four genes involved in early flavonoid biosynthesis before the anthocyanin and flavonol branch point, were measured in seven of these cultivars (Fig. 4f). PAL expression was highest in AmaRosa and Magic Molly, the two cultivars with the highest amounts of total phenolics and lowest in Russet Burbank, the cultivar with the least total phenolics (Fig. 4a). Expression of the early flavonoid pathway genes, including CHS that encodes the first committed enzyme in flavonoid biosynthesis, were generally highest in La Ratte (Fig. 4f), which had the highest amounts of flavonols (Fig. 4b). Other than F3′H, expression of these genes was lowest in the cultivar with the lowest flavonol amounts, Russet Burbank and F3′H expression seemed quite variable.

### *MYB12* expression and flavonol amounts in different organs

Relative to flavonol amounts in some other crops including tomatoes, onions and broccoli^51,52^, tubers had low amounts, which might suggest potato lacks the necessary genes for efficient flavonol synthesis (Figs. 4b and S4). However, flavonol amounts were much higher in leaves and flowers, which are organs exposed to light during growth, in contrast to tubers that develop in the dark (Fig. 4g). This showed that potato has the necessary genes and biosynthetic capacity to synthesize large amounts of flavonols. Flowers had the highest concentration of flavonols, over 700-fold higher than in tubers, and leaves had about 75-fold higher amounts than tubers. FLS catalyzes the committed step in flavonol biosynthesis, and flavonol amounts were consistent with the amount of *FLS* expression in flowers, leaves, and tubers (Fig. 4h). Given these dramatic differences in flavonols, the expression of *StMYB12A*, *B*, and *C* was compared among flowers, leaves and tubers. *StMYB12C* was the most highly expressed of the three MYBs, whereas *MYB12B* had very low expression in all organs (Fig. 4i). *MYB12A* was more highly expressed than *MYB12B*, with expression levels in leaves and tubers similar to that of *MYB12C*, but it had much lower expression in flowers than *MYB12C*.

### Agroinfiltration of *MYB12* in potato leaves

**Fig. 5:**
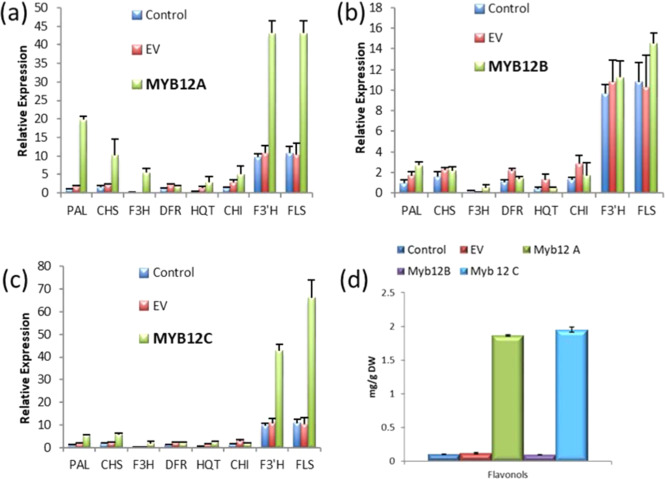
Transient expression of *MYB12A*, *B*, and *C* in potato leaves. Expression of phenylpropanoid structural genes after infiltration of potato leaves with **a** MYB12A, **b** MYB12B, and **c** MYB12C. **d** Flavonol amounts after infiltration with MYB12A, B, or C. The data represents the means ± standard deviation of three biological replicates

The above results provide correlative evidence for a role for StMYB12A and C in potato flavonol regulation. To functionally characterize StMYB12 transcription factors, plant expression constructs containing *MYB12A*, *B*, and *C* were infiltrated into potato leaves to examine the effect of transient expression. Flavonols increased markedly in leaves infiltrated with *MYB12A* and *C* (Fig. 5d) as did chlorogenic acid (Fig. S5). No increase in anthocyanins was observed. Expression of early pathway genes such as *PAL* and flavonoid biosynthesis genes such as *CHS, F3H, F3*′*H*, and *FLS* were increased in leaves infiltrated with *MYB12A* or *C* (Fig. 5a–c). FLS expression was highest in leaves infiltrated with *MYB12C*. In contrast, infiltration with *MYB12B* had little effect on phenylpropanoid gene expression or metabolites.

### StmiR858 negatively regulated flavonol biosynthesis through repressing *MYB12*

An additional potential regulatory mechanism for flavonol biosynthesis is via microRNAs. The microRNA, miR858, is involved in flavonol biosynthesis in Arabidopsis, apple (*Malus domestica*), muskmelon (*Cucumis melo*), and peach (*Prunus persica*)^45–48^. However, this has not been reported in potato. By searching the microRNA database of potato (mpss.danforthcenter.org/dbs), a putative miR858 homolog was located on chromosome 5 of potato (Fig. S6a). The mature sequence of StmiR858 contained 21 nucleotides with only a one nucleotide difference from the peach miR858 (Fig. S6b). The miR858 precursor was predicted to contain 85 nucleotides (Fig. S6c) using the software miRNAfold tanuki.ibisc.univ-evry.fr^53^.

**Fig. 6:**
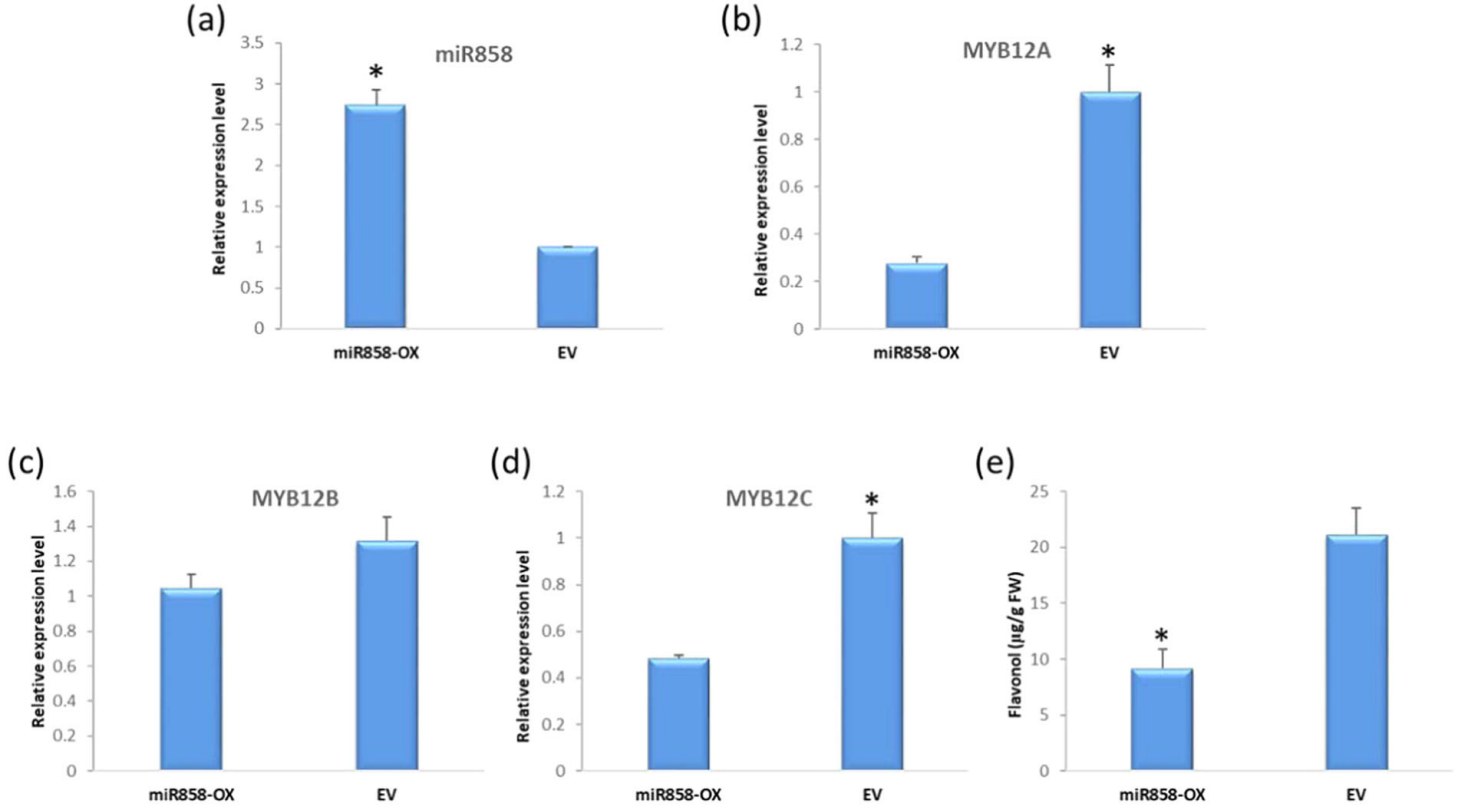
Infiltration of miR858 into potato leaves. **a** Expression level of mature miR858 after infiltration with the overexpressing construct or empty vector. **b**–**d** represent the expression level of MYB12A, B, and C in miR858 and empty pORE-O2 vector infiltrated leaves respectively. **e** Flavonol content in potato leaves after infiltration with and without overexpression of miR858. The data represents the means ± SD (*n* = 3). Asterisks indicates significant difference (*p* < 0.05)

An analysis was then conducted for potential targets of StmiR858 in the potato genome by using psRNATarget (plantgrn.noble.org/psRNATarget/home). The results showed that most of the potential targets are MYB transcription factors and included MYB12A and C (Table 1). To examine whether StmiR858 affected *MYB12* expression and inhibits flavonol biosynthesis, a StmiR858 transient overexpression construct was agroinfiltrated into potato leaves. Infiltration increased miR858 levels ~2.5-fold (Fig. 6a). Expression of *MYB12A* in miR858 infiltrated leaves was 72% lower than the empty vector control (Fig. 6b). *MYB12C* was repressed more than 50% (Fig. 6d). However, a significant difference in *MYB12B* expression was not detected in leaves infiltrated with miR858 (Fig. 6c). Additional evidence *MYB12A* and *C* regulate potato flavonol metabolism and are affected by miR858, was that flavonol concentrations in the miR858 overexpression leaves decreased by ~57% (Fig. 6e). These results suggested that StmiR858 directly repressed *MYB12* to regulate flavonol biosynthesis in potato.

**Table 1:** Potential targets of StmiR858 in potato

Target gene ID	Target start	Target end	Target annotation	Target aligned fragment	Inhibition
PGSC0003DMT400023322	335	355	MYB 12 transcription factor (MYB12C)	UCAGGUAGAACAGACAAUGAG	Cleavage
PGSC0003DMT400014983	289	309	MYBPA1 protein	CCAGGUCGAACAGAUAAUGAA	Cleavage
PGSC0003DMT400005061	2214	2234	Glycosyl transferase family 1 protein	UGAGUUUGAACAGAUAAUGAG	Cleavage
PGSC0003DMT400018841	302	322	MYB 12 transcription factor (MYB12A)	CCAGGUAGAACAGACAAUGAA	Cleavage
PGSC0003DMT400030322	109	129	Hypothetical gene of unknown function	CCAGGACGAACAGACAAUGAA	Cleavage
PGSC0003DMT400018840	268	288	MYB 12 transcription factor	CCAGGUAGAACAGACAAUGAA	Cleavage
PGSC0003DMT400036086	530	550	Protein 3	CCCGGUAGAACAGACAAUGAG	Cleavage
PGSC0003DMT400016394	289	309	Transcription factor MYB102	UCAGGAAGAACAGACAAUGAG	Cleavage
PGSC0003DMT400035505	1726	1746	JmjC domain containing protein	GCAGGUUGAACAAAUAAUGAG	Cleavage

Target genes were predicted using psRNATarget. Gene annotations were obtained from Potato Genome Database (http://solanaceae.plantbiology.msu.edu/pgsc_download.shtml). Of these potential target genes, PGSC0003DMT400023322 and PGSC0003DMT400018841 are MYB12C and MYB12A, respectively

**Fig. 7:**
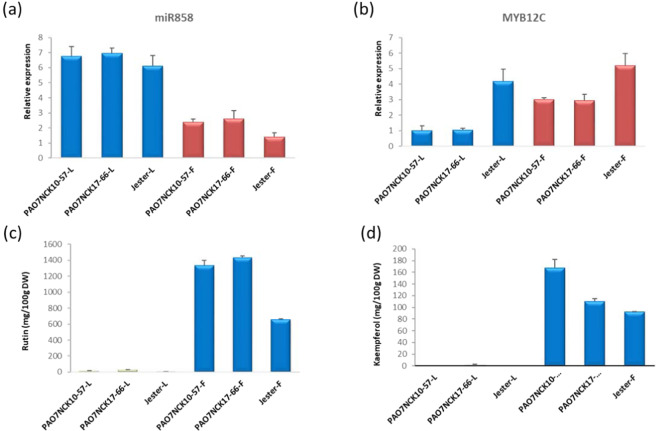
Expression of MYB12C and miR858 in flowers and leaves of potato, and flavonol content. **a** Endogenous expression level of miR858 in potato leaves and flowers from three genotypes. **b** MYB12C endogenous expression levels in leaves and flowers from three potato genotypes. **c** Rutin content. **d** kaempferol content. -L, leaf; -F, flower. PAO7NCK10-57 and PAO7NCK17-66 are genotypes with white flowers, and Jester has purple flowers. The data represents the means ± SD of three biological replicates

The concentration of flavonols in flowers and leaves was determined, along with levels of *MYB12C* and StmiR858. Unlike white flowers, purple potato flowers have high amounts of anthocyanins, which compete with the flavonol branch of the pathway for common precursors. To assess potential differences between white and purple flowers on flavonol metabolism, both were examined. Expression of miR858 was higher in leaves than flowers (Fig. 7a). There was not a notable difference in miR858 expression in leaves from plants with purple flowers versus white flowers. Conversely, *MYB12C* was expressed 2.7 to 4.3-fold higher in flowers than in leaves (Fig. 7b). In particular, white flowers had higher expression of *MYB12C* relative to leaves from the same plants (Fig. 7b). Purple flowers had the lowest expression of miR858, but highest expression of *MYB12C*. Rutin and kaempferol were present in much higher concentrations in flowers than leaves (Fig. 7c, d), which is consistent with the higher expression of miR858 in leaves (Fig. 7a). StmiR858 expression was barely detectable in tubers (data not shown).

## Discussion

One potential explanation for the low amount of flavonols in tubers (Figs. 4b and S4) is that potato lacked the genes necessary for efficient flavonol metabolism. However, the high levels of flavonols in leaves and flowers do not support this explanation (Fig. 4g). Consequently, we sought to better characterize flavonol regulation in potato. While StAN1 affects multiple phenylpropanoid branches including flavonols, its primary target is anthocyanins. Additional factors must regulate potato flavonol metabolism. MYB12 regulates flavonol biosynthesis in Arabidopsis and tomato^28,54^. Transient overexpression of StMYB12A or C increased expression of flavonoid pathway genes including *CHS*, which encodes the first committed enzyme in flavonoid biosynthesis, *CHI*, which converts chalcone into naringenin, the precursor for all flavonoids, and *F3H*, which synthesizes dihydroflavonols, the direct substrate for FLS^14,55^.

The large increase in *FLS* expression and flavonol amounts in leaves infiltrated with StMYB12 A and C (Fig. 5) supports these MYBs being key regulators of potato flavonol metabolism, but do not preclude a role for additional, as yet unidentified, MYBs to regulate potato flavonols. In addition to flavonols, MYB12 A and C increased *PAL* expression and general phenylpropanoid metabolism, similar to what we observed previously with StAN1, the overexpression of which was not specific to the anthocyanin pathway^20^. Whereas overexpression of MYB12 had some overlapping effects to what we previously reported for StAN1, there were also differences. StAN1 infiltration in leaves increased anthocyanin synthesis, but StMYB12 infiltration did not.

Different phenylpropanoid regulatory mechanisms appear to be involved in different potato organs. In potato leaves, the anthocyanin pathway may be more tightly regulated than the flavonol branch. StAN1 and StMYB12 overexpression both increased activity of the overall general pathway and amounts of various leaf phenylpropanoids including flavonols, but only AN1 increased anthocyanins^50^. Thus, in potato leaves a consequence of increased general phenylpropanoid activity was increased flavonols but not anthocyanins. This is consistent with what was seen in tobacco leaves, where overexpressing AtPAL2 caused a two-fold increase in chlorogenic acid, a 5-fold increase in flavonols but no increase in anthocyanins^56^. In contrast, a general increase in tuber phenylpropanoid metabolism does not lead to increased flavonols. In potatoes with red or purple flesh, extremely high amounts of anthocyanins and various non-anthocyanin phenylpropanoids are present showing the pathway is very active, yet this is not enough to lead to elevated amounts of flavonols (Fig. 4). This is consistent with previous work in which potato phenylpropanoid metabolism was rerouted by suppressing hydroxycinnamic acid synthesis, and anthocyanins but not flavonols increased in rerouted purple tubers, whereas both increased in flowers^20^. Collectively, such data suggests high activity of the general phenylpropanoid pathway leads to elevated flavonols in leaves but not tubers, and flavonol synthesis is more tightly regulated in tubers than in leaves or flowers.

The effect of these R2R3 MYBs on phenylpropanoids outside of the specific anthocyanin or flavonol branches they target may be explained in part by coordinate regulation of the pathway^57^ and the need to increase PAL expression, which is rate limiting for the pathway, so that increased carbon flow is available to supply precursors for downstream anthocyanin and flavonol biosynthesis^14^. An additional explanation for why StMYB12 and StAN1 have a broad effect on phenylpropanoid metabolism, may be their interaction with sucrose.

Besides its role in primary metabolism, sucrose acts as a signal molecule that is responsive to environmental factors including light and temperature, including for phenylpropanoid metabolism^58–60^. The regulatory role of sucrose, which involves a mitogen-activated protein kinase (MAPK) signaling cascade, has been especially studied in plant anthocyanin metabolism, including grapes and Arabidopsis^61–63^. Previous work with potato showed correlations between sucrose and phenylpropanoid amounts during development and environmental responses^19^.

**Fig. 8:**
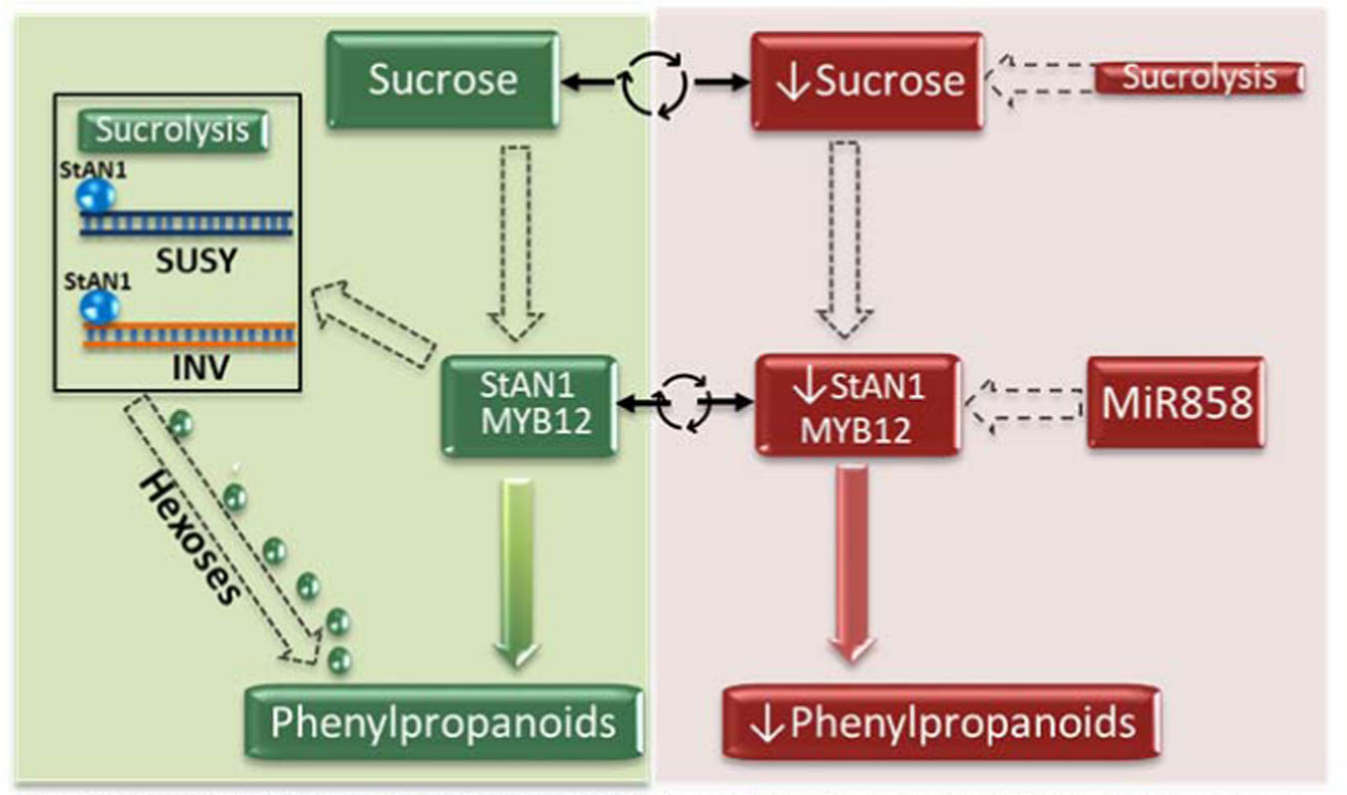
Proposed model for phenylpropanoid regulation in potato. Sucrose has both a regulatory role and a metabolic role by supplying hexoses that can be channeled to phenylpropanoid biosynthesis. In the left green panel, sucrose increases MYB expression, which in turn increases phenylpropanoid metabolism. StAN1 concurrently increases expression of sucrolytic genes that generate hexoses to feed increased phenylpropanoid metabolism. In the right red panel, phenylpropanoid synthesis decreases either due to reduced StAN1 expression resulting from falling sucrose levels caused by StAN1 induced sucrolysis, or from increased expression of MiR858, a negative regulator. Sucrose concentrations and MYB expression form a self-regulatory cycle, where sucrose promotes MYB expression but MYB expression promotes sucrolysis. The circular arrows connecting sucrose in the two panels, indicate that the concentration of sucrose cycles and influences phenylpropanoid metabolism. Green indicates conditions that increase phenylpropanoid metabolism and red conditions that do not. Solid arrows indicate direct effects on phenylpropanoid regulation and dashed arrows indirect

Previous work showed StAN1 overexpression decreased amounts of sucrose and hypothesized that this may be a consequence of putative MYB binding sites in the promoters of sucrolytic genes^50^. Here, we showed that purified StAN1 directly interacts with promoter fragments from sucrolytic genes (Fig. 2), and that the promoters of MYB12A and C each had four sucrose responsive elements (Table S2). Why MYB12B had little impact on flavonol metabolism is not clear, but it is interesting to note its lack of sucrose responsive elements. These data further support a model (Fig. 8) in which sucrose and MYBs are involved in a self-feedback loop to mediate phenylpropanoid expression, and may in part explain why both MYB12 and AN1 overexpression tend to increase general phenylpropanoid metabolism.

In this proposed model, upregulated MYB expression increases sucrose catabolism. This may allow a finely tuned regulatory cycle in which sucrose amounts are balanced between promoting MYB expression and channeling hexoses into phenylpropanoid metabolism via the glycolytic and shikimate pathways. By promoting sucrose catabolism, MYB expression would be predicted to downregulate its own expression by decreasing the supply of sucrose if carbohydrate metabolism does not maintain sufficient levels of sucrose. This interaction between MYBs, sucrolytic genes, and sucrose suggests a mechanistic link between phenylpropanoid metabolism and starch-sucrose metabolism. Carbohydrate metabolism in potato, including starch-sucrose interconversion, is finely regulated^64^, and this linkage may allow an additional fine mechanism of control for tuber phenylpropanoid regulation.

This work also showed miR858 affects potato flavonol metabolism, further indicating the complexity of flavonol regulation. MiRNAs are involved in a variety of processes, such as plant growth and development, host–pathogen interactions, apoptosis and phenylpropanoid metabolism^65–67^. MiR828 is involved in phenylpropanoid metabolism and interacts with *MYB* transcription factors such as MYB113, PAP1, and PAP2 to coordinate anthocyanin biosynthesis in *Pinus resinosa*^68^. Overexpression of miR828 reduced anthocyanin levels in Arabidopsis and led to early flowering and downregulation of MYB transcription factors related to flavonoid biosynthesis^69^. However, others suggest miR828 is a positive regulator of anthocyanin synthesis in potato and grapes^44,70^. Similar contradictory findings have been reported about miR858, where it has been reported as a negative regulator of anthocyanins in Kiwi fruit and tomato^71,72^ but a positive regulator in Arabidopsis^73^.

Based on evidence linking it to flavonol regulation in other plants, miR858 seemed a logical candidate in potato^45–48^. Unlike its effect on MYB12A and C, StmiR858 overexpression did not affect MYB12B, which is consistent with the other data that did not support a role for MYB12B in potato flavonol metabolism. Moreover, flavonol content was much higher in flowers than in leaves, and endogenous StmiR858 levels in leaves were significantly higher than that in flowers, which would be expected if miR858 was a negative regulator. However, unlike in leaves or flowers, barely detectable levels of miR858 were present in tubers (data not shown). So, while StmiR858 was involved in flavonol regulation, it did not appear to be the explanation for why flavonols are present in high amounts in potato flowers and leaves, but not in tubers. Additional research is needed to determine if MiR858 affects additional genes, such as other candidates in Table 1.

Additional, unknown regulatory mechanisms must be involved in the regulation of tuber flavonol biosynthesis. Given the high amount of flavonols in flowers, the lack of necessary genes in potato is not an explanation, nor is an inability of tubers to synthesize flavonol precursors, given that tubers can have very high amounts of anthocyanins that share immediate precursors with flavonols. One possible mechanism might involve light, given that potato organs that develop in the light (leaves and flowers) had up to 700-fold higher amounts of flavonols than organs that develop in the dark, (tubers) and numerous light-responsive elements were present in the MYB12 promoters (Fig. 4g and Table S3). Light is widely known to promote flavonol synthesis in plants^74,75^ and has also been shown to induce expression of miR858 in plants^69,76^, suggesting microRNAs could be another mechanism that transduce environmental signals to mediate potato phenylpropanoid metabolism.

In summary, the direct interaction of StAN1 with promoter sequences of multiple sucrolytic genes and the presence of sucrose responsive elements in the promoters of MYB12A and C support an important role for sucrose in regulating potato phenylpropanoids. MYB12A and C were positive regulators of FLS, the expression of which appears to be a key determinant of flavonol concentrations in potato. StmiR858 functioned as a negative regulator of flavonol metabolism and adds another layer of complexity to potato phenylpropanoid regulation. The presence of various environmentally responsive *cis*-acting elements in the StAN1 and StMYB12 promoters, along with the proposed MYB-sucrose regulatory loop suggest mechanisms for how potato phenylpropanoid amounts are modulated. Additional studies are needed to delineate why tubers have much lower flavonol amounts than leaves, and to devise ways to selectively increase individual phenylpropanoid branches without increasing others. Understanding the regulatory mechanisms that control phenylpropanoid metabolism in potato will facilitate developing plants with optimized phenylpropanoid profiles for human health and crop performance.

## Materials and methods

### Isolation of MYB12 homologs from potato and phylogenetic analysis

MYB12 sequences from Arabidopsis and tomato were blasted in potato genome sequence databases to identify putative potato homologs^77^. Protein blast searches showed three putative MYB12 homologs in potato. *StMYB12A*, *B*, and *C* were amplified from potato cDNA using gene specific primers designed to isolate the full-length cDNA. The amplified gene fragments were cloned into TOPO T/A cloning vector and transformed in *E. coli*. Constructs were verified by sequencing. Sequences were aligned using Clustal omega alignment software and a phylogenetic tree was constructed with neighbor joining method using TOL^78^.

### Gene expression analysis

Gene expression was analyzed by qRT-PCR analysis. PCR was carried out using cDNA prepared from 2 µg of total RNA and further diluting the cDNA ten times. Specific primers were used for expression of different genes (Table S4). PCR reactions were setup in 10 µl reactions with 3 µl diluted cDNA, 1 µl forward primer, 1 µl reverse primer, and 5 µl SYBR Green mix. Samples had a 2 min preincubation at 95 °C followed by 40 cycles of 10 s denaturation at 95 °C, 15 s annealing at 60 °C, and 25 s extension at 72 °C, in a LightCycler 480 (Roche). Relative expression of the genes was calculated by the ΔΔCT method by normalizing the expression levels of target genes to the expression mean of the housekeeping gene actin. Delta CT = CT value of target gene—CT value of housekeeping gene (Actin). Expression = 2^(-delta delta CT)^. The expression level of miR858 was detected with stem-loop RT-PCR method^79^.

### Agrobacterium infiltration studies

Full-length coding sequences of the transcription factors St*MYB12 A, B, C*, and *AN1* were cloned into the modified binary vector pOREO2 with a 35S promoter^80^. The artificial miR858 expression plasmid was constructed following the protocol on website wmd3.weigelworld.org^81^. The pRS300 plasmid was used to generate artificial miR858. Positive clones were confirmed by PCR and sequencing. An infiltration approach was adopted based on published studies^82,83^. A single positive colony was cultured in 5 ml of LB medium overnight and used to inoculate 50 ml of medium. Cells were harvested and adjusted to O.D. 0.5 in 10 mM MgCl_2_ containing 100 μM acetosyringone. Cultures were then diluted (1:1) with the gene silencing suppression vector p19TBSV of tomato bushy stunt virus^37^. Samples were infiltrated into leaves of 4-week-old potato plants (Solanum tuberosum cv. La Ratte). Leaves were harvested at 2 days of post-infiltration. Empty vector was used as control along with non-infiltrated samples.

### Phenylpropanoid quantification

Phenylpropanoids were measured in light and dark grown organs (leaves, flowers, tubers). Total phenolics were extracted from 50 mg freeze-dried samples with a total of 1.5 ml of 50% methanol, 1 mM EDTA, and 2.5% metaphosphoric acid. Total phenolics were quantitated as gallic acid equivalents (mg gallic acid/g extract) using the FC method^84^. Reactions were measured at 755 nm using a Synergy 2 Multi-Mode microplate reader (BioTek Instruments). Flavonols were analyzed by using HPLC-MS, with 20 μl of sample injected onto an Agilent 1200 HPLC equipped with an Onyx 100 × 4.6 mm column (Phenomenex) at 35 °C with a flow rate of 1 ml min^−1^ and a gradient elution of 0–1 min 100% A, 1–9 min 0–30% B, 9–10.5 min 30% B, 10.5–14 min 35–65% B, 14–16 min at 65–100% B, 16–16.5 min 100% B (Buffer A: 10 mM formic acid pH 3.5 with NH4OH; Buffer B: 100% methanol with 5 mM ammonium formate as described^50^). Flavonols were quantified as rutin equivalents.

### Heterologous expression of StAN1 protein in *E. coli*

The potato AN1 cDNA comprising of start codon and stop codon was amplified with forward and reverse primers having *BamHI* and *SacI* sites, respectively. Amplified product was cloned first in T/A cloning vector. After sequence confirmation, the full-length cDNA fragment was ligated into the pET28a vector (Novagen). These constructs were transformed into the host strain *E*. *coli* (Shuffle cells) for IPTG-induced expression. Recombinant his-tagged StAN1 was obtained from cells grown in 200 ml Luria Broth with shaking at 37 °C until an O.D. of 0.4. IPTG (isopropyl-β-d-thiogalactopyranoside) was added to 0.4 mM, the incubation continued for 3 h, after which cells were harvested by centrifugation at 5000×*g* for 5 min at 4 °C. The pellet was resuspended in 5 ml cold protein extraction buffer (20 mM Tris-Cl (pH-8.0), 200 µg/ml of lysozyme, 150 mM NaCl and 20 µg/ml DNase1, 5 mM imidazole and 2 M urea). Protein was isolated using a repetitive freeze thaw method and purified on a NTA column (Qiagen) at 4 °C according to manufacturer instructions, with stepwise imidazole elution at 25, 50, 100, and 200 mM. The eluted fractions were analyzed on SDS page^85^.

### Isolation of promoter fragments for *SUSY1*, *SUSY4*, *INV1*, and *INV2*

Cultivar Purple Majesty genomic DNA was used to isolate promoter fragments (~1.5 kb) of SUSY1, SUSY4, INV1, and INV2. Specific primers were designed from promoter sequences obtained from the potato genome database^77^. Isolated promoter fragments were sequenced and MYB binding consensus sequences identified within them (Fig. S1). Cloned fragments of SUSY1, SUSY4, INV1, and INV2 were used as templates, and nested PCR was carried out using specific primers designed to amplify 100–150 bp regions containing multiple MYB binding sequences. The amplified regions were cloned and sequenced to confirm their identity.

### Electrophoretic mobility shift assay for StAN1 and sucrolytic gene promoters

Fifty nanogram of amplified DNA fragments (*SUSY1, SUSY4, INV1*, and *INV2*) were end-labeled with biotin. A LightShift chemiluminescent EMSA kit was used according to directions (Thermo Scientific) and blots were photographed with a CCD camera. Two microgram of purified protein was used for binding. For competition assays a 5-fold or 50-fold excess of unlabeled transcript was used.

## Supplementary information

supplemental material

## Supplementary information

**Supplementary Information** The online version contains supplementary material available at (10.1038/s41438-021-00463-9).
