# Supplementary material for: R2R3-MYB transcription factors, StmiR858 and sucrose mediate potato flavonol biosynthesis
Source: Hortic Res. 2021 Feb 1;8:25. doi: 10.1038/s41438-021-00463-9 (PMC7847999; doi:10.1038/s41438-021-00463-9)
Supplement: Supplementary file 1 — supplemental material [file 41438_2021_463_MOESM1_ESM.docx]

**Supporting Information**

**Figure S1** (a) Sucrolytic genes *SUSY1*, *SUSY4*, *INV1* and *INV2* have multiple MYB binding domains in their promoters. (b) Graphical representation of the SUSY1 promoter. MYBCore–CNGTTR, MYB Plant-MACCWAMC, MYB PZM- CCWACC, MYB consensus- CANNTG

**Figure S2.** SDS-PAGE analysis of overexpressed His-tagged StAN1 fusion protein. Samples were analyzed on 10% SDS-PAGE with Coomassie blue staining. Lane M, Protein size markers, Lane 1, uninduced crude extract; Lane 2, induced crude extract; Lane3, Ni-NTA column flow-through; Lane 4-8, Eluted protein (StAn1) using 10mM, 25mM, 50mM, 100mM, and 200mM imidazole respectively. Positions and size of marker and induced protein are shown.

**Figure S3.** Multiple MYB sequence alignment using Clustal Omega (<https://www.ebi.ac.uk/Tools/msa/clustalo/>). The alignment result was further reformatted with Mview (<https://www.ebi.ac.uk/Tools/msa/mview/>). At, *Arabidopsis thaliana*; Gt, *Gentiana trifloral*; Md, *Malus domestica*; Sl, *Solanum lycopersicum*; St, *Solanum tuberosum*; Vv, *Vitis vinifera*; Zm, *Zea mays*. Cov, percent coverage; Pid, percent identity.

**Figure S4.** Flavonol content and MYB12C expression in 10 different potato genotypes. (a) Kaempferol concentration; (b) Rutin concentration; (c) Expression of *MYB12C*; (d) Correlation between *MYB12C* level and kaempferol concentration, p < 0.001; (e) Correlation between *MYB12C* and rutin concentration in tubers, p < 0.001. At least 3 biological replicates were measured, error bars illustrate standard deviation.

**Figure S5.** Increase in chlorogenic acid (CGA) in response to infiltration with MYB12s.

**Figure S6.** Identification of miR858 in potato. (a) Locus of miR858 in potato genome. (b) Mature sequences of miR858 in different plants. AT, *Arabidopsis thaliana*; MD, *Malus domestica*; CM, *Cucumis melo*; PP, *Prunus persica*; ST, *Solanum tuberosum*. (c) Predicted secondary structure of miR858 precursor in potato, the mature sequence is highlighted in purple. Potential targets of miR858 are shown in Table 1.

**Table S1.** BLAST results using protein sequences of StMYB12A, B and C with the Arabidopsis protein database. Identity, similarity and score indicate the homology between the query genes and AtMYB12.

**Table S2.** Sucrose responsive elements in promoters of MYB12A, B and C.

**Table S3.** Cis-acting elements in the MYB12 A,B and C promoters.

**Table S4.** Primers used in this study.

**
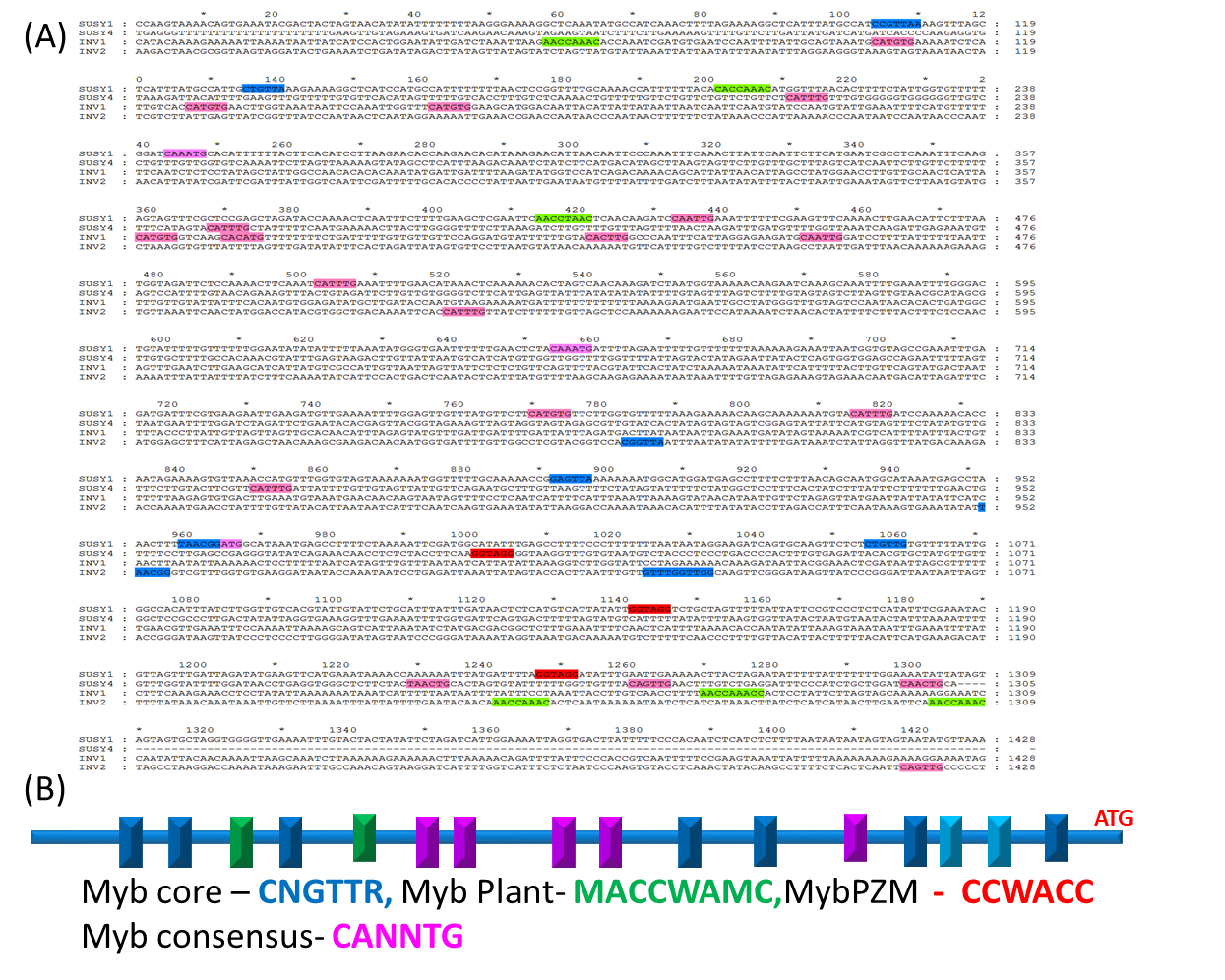
**

**Figure S1.** (a) Sucrolytic genes *SUSY1*, *SUSY4*, *INV1* and *INV2* have multiple MYB binding domains in their promoters. (b) Graphical representation of the SUSY1 promoter. MYBCore–CNGTTR, MYB Plant-MACCWAMC, MYB PZM- CCWACC, MYB consensus- CANNTG

**Figure S2.** SDS-PAGE analysis of overexpressed His-tagged StAN1 fusion protein. Samples were analyzed on 10% SDS-PAGE with Coomassie blue staining. Lane M, Protein size markers, Lane 1, uninduced crude extract; Lane 2, induced crude extract; Lane3, Ni-NTA column flow-through; Lane 4-8, Eluted protein (StAn1) using 10mM, 25mM, 50mM, 100mM, and 200mM imidazole respectively. Positions and size of marker and induced protein are shown.

**Figure S3.** Multiple MYB sequence alignment using Clustal Omega (<https://www.ebi.ac.uk/Tools/msa/clustalo/>). The alignment result was further reformatted with Mview (<https://www.ebi.ac.uk/Tools/msa/mview/>). At, *Arabidopsis thaliana*; Gt, *Gentiana trifloral*; Md, *Malus domestica*; Sl, *Solanum lycopersicum*; St, *Solanum tuberosum*; Vv, *Vitis vinifera*; Zm, *Zea mays*. Cov, percent coverage; Pid, percent identity.


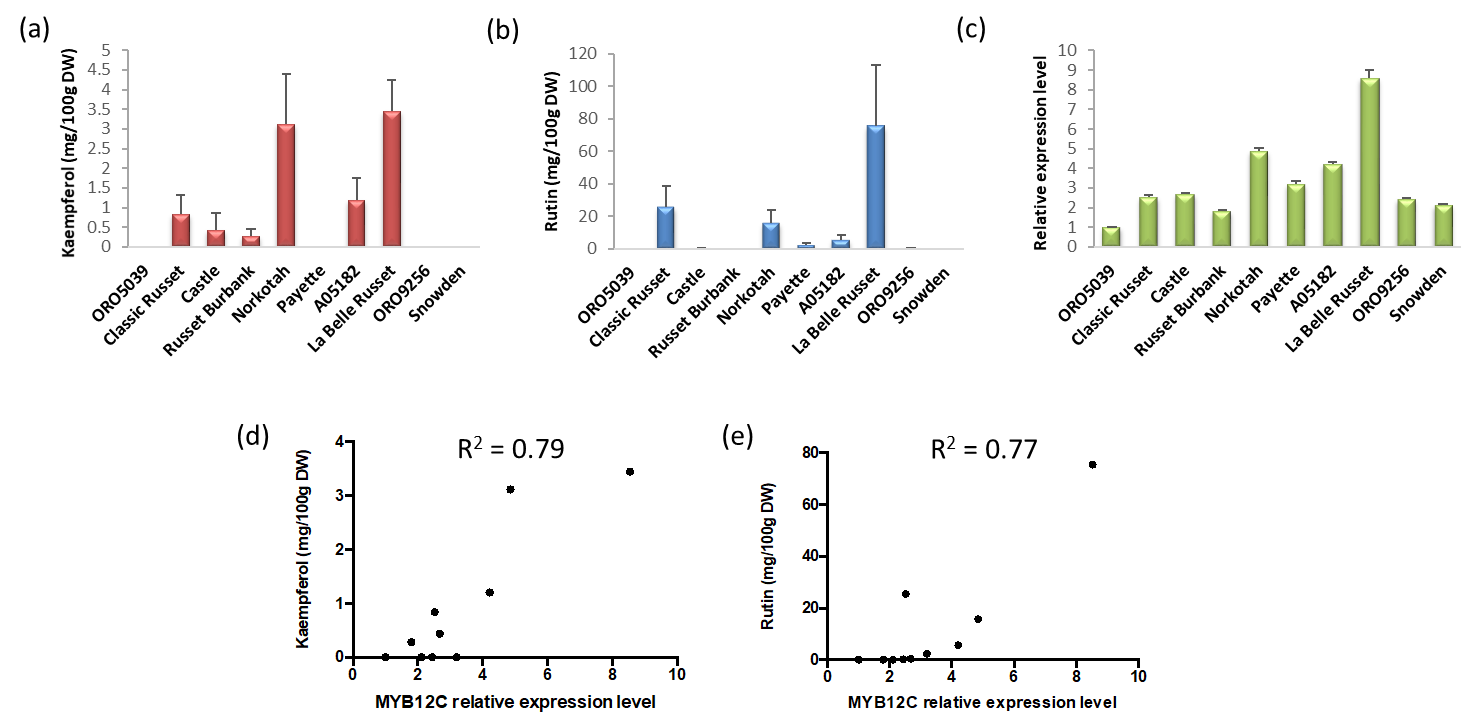


**Figure S4.** Flavonol content and MYB12C expression in 10 different potato genotypes. (a) Kaempferol concentration; (b) Rutin concentration; (c) Expression of *MYB12C*; (d) Correlation between *MYB12C* level and kaempferol concentration, p < 0.001; (e) Correlation between *MYB12C* and rutin concentration in tubers, p < 0.001. At least 3 biological replicates were measured, error bars illustrate standard deviation.


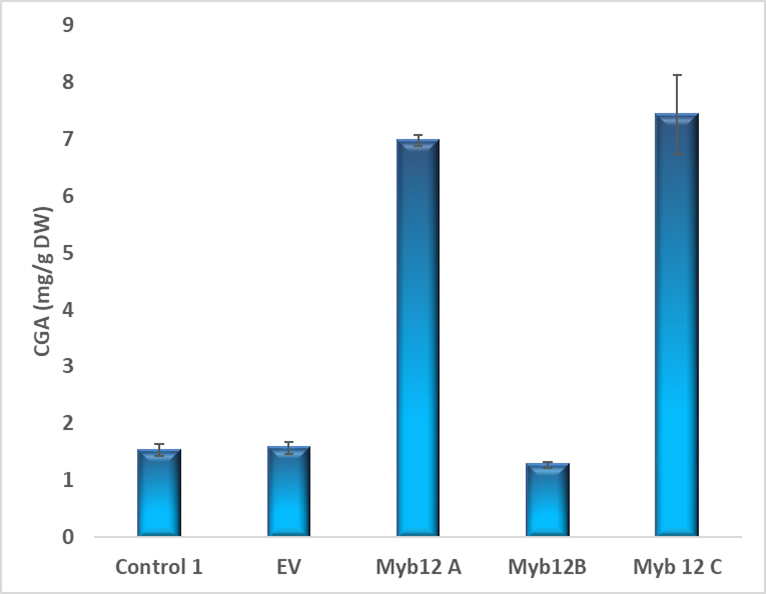


**Figure S5.** Increase in chlorogenic acid (CGA) in response to infiltration with MYB12s.


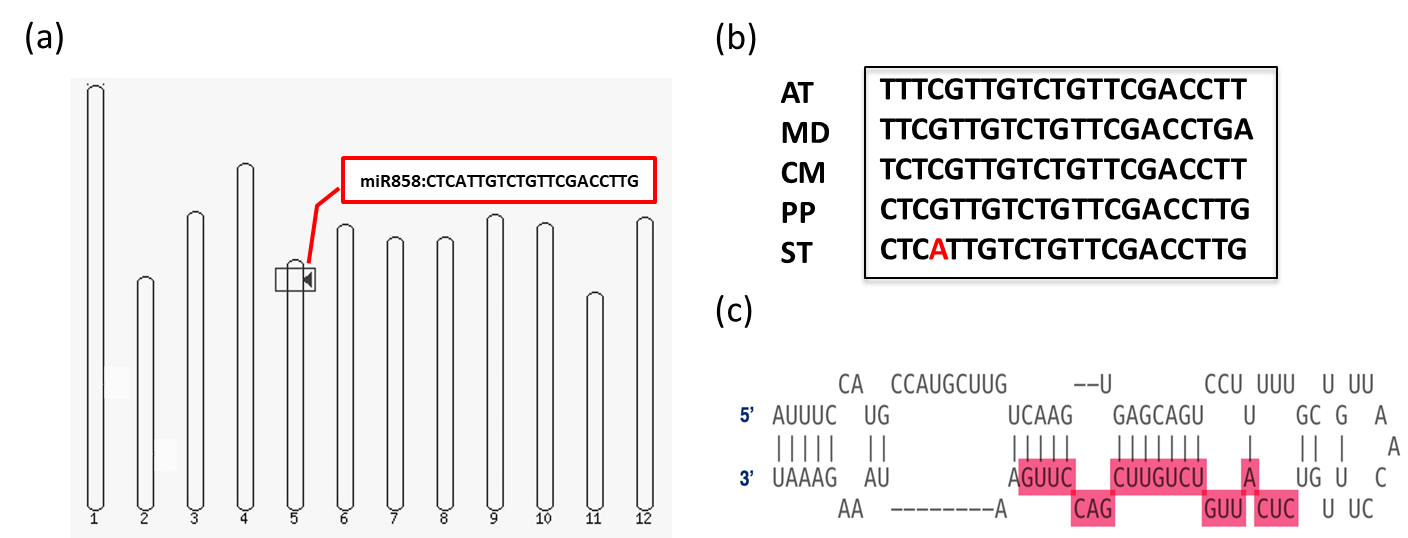


**Figure S6.** Identification of miR858 in potato. (a) Locus of miR858 in potato genome. (b) Mature sequences of miR858 in different plants. AT, *Arabidopsis thaliana*; MD, *Malus domestica*; CM, *Cucumis melo*; PP, *Prunus persica*; ST, *Solanum tuberosum*. (c) Predicted secondary structure of miR858 precursor in potato, the mature sequence is highlighted in purple. Potential targets of miR858 are shown in Table 1.

**Compared to AtMYB12**


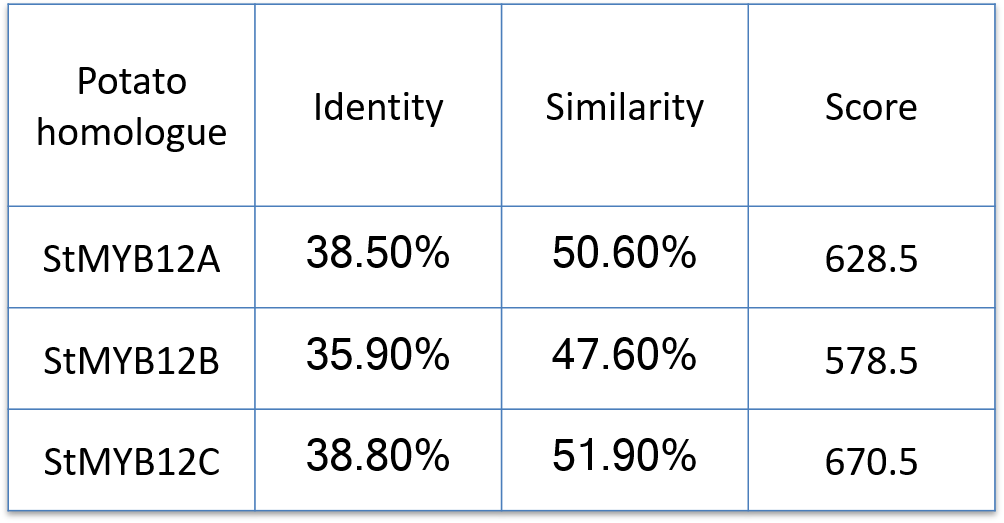


**Table S1.** BLAST results using protein sequences of StMYB12A, B and C with the Arabidopsis protein database. Identity, similarity and score indicate the homology between the query genes and AtMYB12.

**
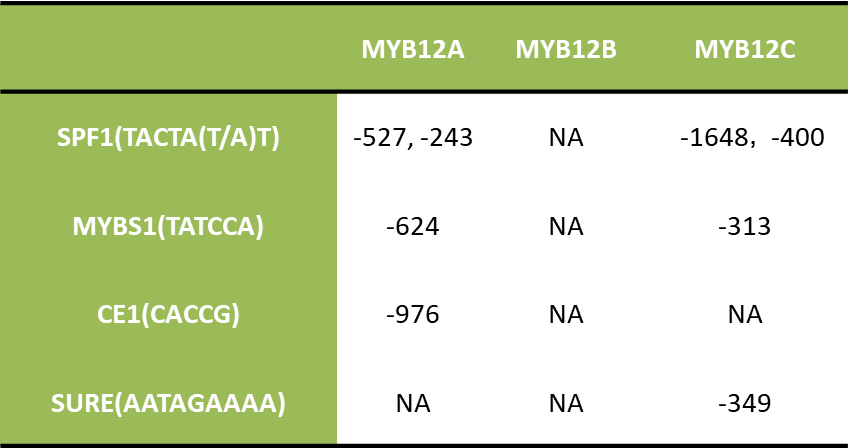
**

**Table S2.** Sucrose responsive elements in promoters of MYB12A, B and C.

| **Gene** | **Motif** | **Position** | **Strand** | **Hit sequence** |
| --- | --- | --- | --- | --- |
|  |  |  |  |  |
| **MYB12A** | GT1CONSENSUS | 44 | - | GAAAAA |
|  | GT1CONSENSUS | 63 | + | GATAAT |
|  | GT1CONSENSUS | 89 | - | GAAAAT |
|  | GT1CONSENSUS | 145 | + | GAAAAA |
|  | GT1CONSENSUS | 300 | + | GAAAAA |
|  | GT1CONSENSUS | 511 | + | GATAAT |
|  | GT1CONSENSUS | 597 | - | GGAAAA |
|  | GT1CONSENSUS | 686 | + | GATAAA |
|  | GT1CONSENSUS | 693 | + | GAAAAT |
|  | GT1CONSENSUS | 728 | - | GAAAAA |
|  | GT1CONSENSUS | 759 | + | GGTAAA |
|  | GT1CONSENSUS | 770 | + | GAAAAA |
|  | GT1CONSENSUS | 804 | - | GAAAAA |
|  | GT1CONSENSUS | 811 | - | GAAAAA |
|  | GT1CONSENSUS | 908 | + | GAAAAT |
|  | GT1CONSENSUS | 971 | - | GAAAAT |
|  | GT1CONSENSUS | 1247 | + | GAAAAA |
|  | GT1CONSENSUS | 1405 | + | GAAAAA |
|  | GT1CONSENSUS | 1515 | + | GAAAAT |
|  | GT1CONSENSUS | 1541 | + | GATAAA |
|  | GT1CONSENSUS | 1590 | + | GAAAAT |
|  | GT1CONSENSUS | 1717 | - | GAAAAT |
|  | GT1CONSENSUS | 1718 | - | GGAAAA |
|  | GT1CONSENSUS | 1784 | + | GGTAAA |
|  | GT1CONSENSUS | 1843 | + | GAAAAA |
|  | GT1CONSENSUS | 1850 | + | GAAAAA |
|  | SORLIP1AT | 1 | - | GCCAC |
|  | SORLIP1AT | 448 | - | GCCAC |
|  | SORLIP1AT | 455 | + | GCCAC |
|  | SORLIP1AT | 1034 | + | GCCAC |
|  | SORLIP1AT | 1393 | + | GCCAC |
|  | SORLIP1AT | 1 | - | GCCAC |
|  | SORLIP1AT | 448 | - | GCCAC |
|  | SORLIP1AT | 455 | + | GCCAC |
|  | SORLIP1AT | 1034 | + | GCCAC |
|  | SORLIP1AT | 1393 | + | GCCAC |
|  | GATA Box | 63 | + | GATA |
|  | GATA Box | 114 | - | GATA |
|  | GATA Box | 268 | + | GATA |
|  | GATA Box | 270 | - | GATA |
|  | GATA Box | 511 | + | GATA |
|  | GATA Box | 686 | + | GATA |
|  | GATA Box | 826 | + | GATA |
|  | GATA Box | 1178 | - | GATA |
|  | GATA Box | 1274 | + | GATA |
|  | GATA Box | 1297 | - | GATA |
|  | GATA Box | 1378 | + | GATA |
|  | GATA Box | 1541 | + | GATA |
|  | GATA Box | 1935 | - | GATA |
|  | GATA Box | 1985 | - | GATA |
|  | GATA Box | 1992 | - | GATA |
|  | IBOXCORE | 63 | + | GATAA |
|  | IBOXCORE | 511 | + | GATAA |
|  | IBOXCORE | 686 | + | GATAA |
|  | IBOXCORE | 1541 | + | GATAA |
|  | IBOXCORE | 1984 | - | GATAA |
|  | BOXIIPCCHS | 767 | + | ATAGAA |
|  | BOXIIPCCHS | 1043 | - | ATAGAA |
|  | BOXIIPCCHS | 1932 | - | ATAGAA |
| **MYB12B** | GT1CONSENSUS | 199 | + | GAAAAT |
|  | GT1CONSENSUS | 307 | - | GATAAA |
|  | GT1CONSENSUS | 368 | + | GGAAAT |
|  | GT1CONSENSUS | 520 | - | GAAAAT |
|  | GT1CONSENSUS | 593 | - | GAAAAA |
|  | GT1CONSENSUS | 901 | - | GAAAAT |
|  | GT1CONSENSUS | 920 | - | GAAAAT |
|  | GT1CONSENSUS | 936 | + | GAAAAA |
|  | GT1CONSENSUS | 962 | + | GGAAAA |
|  | GT1CONSENSUS | 1047 | + | GGAAAA |
|  | GT1CONSENSUS | 1090 | + | GGTAAA |
|  | GT1CONSENSUS | 1114 | + | GGAAAA |
|  | GT1CONSENSUS | 1341 | + | GGTAAA |
|  | GT1CONSENSUS | 1347 | - | GAAAAA |
|  | GT1CONSENSUS | 1360 | + | GAAAAA |
|  | GT1CONSENSUS | 1391 | + | GGAAAA |
|  | GT1CONSENSUS | 1449 | + | GGTAAT |
|  | GT1CONSENSUS | 1500 | - | GGTAAA |
|  | GT1CONSENSUS | 1531 | - | GGTAAA |
|  | GT1CONSENSUS | 1764 | + | GATAAA |
|  | GT1CONSENSUS | 1944 | - | GAAAAA |
|  | GT1CONSENSUS | 1954 | + | GGAAAT |
|  | GT1CONSENSUS | 1991 | + | GAAAAT |
|  | SORLIP1AT | 479 | + | GCCAC |
|  | SORLIP1AT | 668 | - | GCCAC |
|  | SORLIP1AT | 1603 | + | GCCAC |
|  | SORLIP1AT | 1861 | - | GCCAC |
|  | SORLIP1AT | 479 | + | GCCAC |
|  | SORLIP1AT | 668 | - | GCCAC |
|  | SORLIP1AT | 1603 | + | GCCAC |
|  | SORLIP1AT | 1861 | - | GCCAC |
|  | GATA Box | 36 | + | GATA |
|  | GATA Box | 246 | - | GATA |
|  | GATA Box | 273 | + | GATA |
|  | GATA Box | 309 | - | GATA |
|  | GATA Box | 1764 | + | GATA |
|  | GATA Box | 1891 | - | GATA |
|  | GATA Box | 1959 | - | GATA |
|  | GT1 Box | 746 | + | GGTTAA |
|  | GT1 Box | 801 | + | GGTTAA |
|  | GT1 Box | 1866 | + | GGTTAA |
|  | IBOXCORE | 308 | - | GATAA |
|  | IBOXCORE | 1764 | + | GATAA |
|  | BOXIIPCCHS | 407 | - | ATAGAA |
|  | BOXIIPCCHS | 442 | - | ATAGAA |
|  | BOXIIPCCHS | 1541 | - | ATAGAA |
|  | BOXIIPCCHS | 1995 | + | ATAGAA |
| **MYB12C** | GT1CONSENSUS | 196 | - | GAAAAT |
|  | GT1CONSENSUS | 197 | - | GGAAAA |
|  | GT1CONSENSUS | 372 | + | GAAAAT |
|  | GT1CONSENSUS | 394 | - | GATAAA |
|  | GT1CONSENSUS | 525 | + | GGAAAT |
|  | GT1CONSENSUS | 569 | - | GGTAAA |
|  | GT1CONSENSUS | 617 | + | GAAAAA |
|  | GT1CONSENSUS | 633 | - | GAAAAT |
|  | GT1CONSENSUS | 705 | + | GAAAAA |
|  | GT1CONSENSUS | 758 | - | GAAAAT |
|  | GT1CONSENSUS | 909 | + | GGAAAA |
|  | GT1CONSENSUS | 910 | + | GAAAAA |
|  | GT1CONSENSUS | 1170 | - | GAAAAA |
|  | GT1CONSENSUS | 1207 | + | GATAAA |
|  | GT1CONSENSUS | 1227 | + | GAAAAT |
|  | GT1CONSENSUS | 1428 | + | GGAAAA |
|  | GT1CONSENSUS | 1429 | + | GAAAAT |
|  | GT1CONSENSUS | 1636 | + | GAAAAT |
|  | GT1CONSENSUS | 1668 | - | GATAAT |
|  | GT1CONSENSUS | 1778 | + | GAAAAA |
|  | GT1CONSENSUS | 1809 | - | GGTAAT |
|  | GT1CONSENSUS | 1823 | - | GATAAA |
|  | GT1CONSENSUS | 1977 | - | GAAAAT |
|  | G Box | 763 | - | TACGTG |
|  | GATA Box | 21 | + | GATA |
|  | GATA Box | 150 | - | GATA |
|  | GATA Box | 178 | - | GATA |
|  | GATA Box | 189 | - | GATA |
|  | GATA Box | 396 | - | GATA |
|  | GATA Box | 507 | + | GATA |
|  | GATA Box | 724 | + | GATA |
|  | GATA Box | 859 | - | GATA |
|  | GATA Box | 1207 | + | GATA |
|  | GATA Box | 1438 | + | GATA |
|  | GATA Box | 1449 | + | GATA |
|  | GATA Box | 1478 | + | GATA |
|  | GATA Box | 1670 | - | GATA |
|  | GATA Box | 1816 | - | GATA |
|  | GATA Box | 1825 | - | GATA |
|  | IBOXCORE | 395 | - | GATAA |
|  | IBOXCORE | 1207 | + | GATAA |
|  | IBOXCORE | 1669 | - | GATAA |
|  | IBOXCORE | 1824 | - | GATAA |
|  | BOXIIPCCHS | 870 | + | ATAGAA |
|  | BOXIIPCCHS | 940 | - | ATAGAA |
|  | BOXIIPCCHS | 998 | - | ATAGAA |
|  | BOXIIPCCHS | 1633 | + | ATAGAA |
| **Table S3.** Light-responsive cis-acting elements in the MYB12A, B and C promoters. | | | | |

|  |  |  |  |  |
| --- | --- | --- | --- | --- |

**
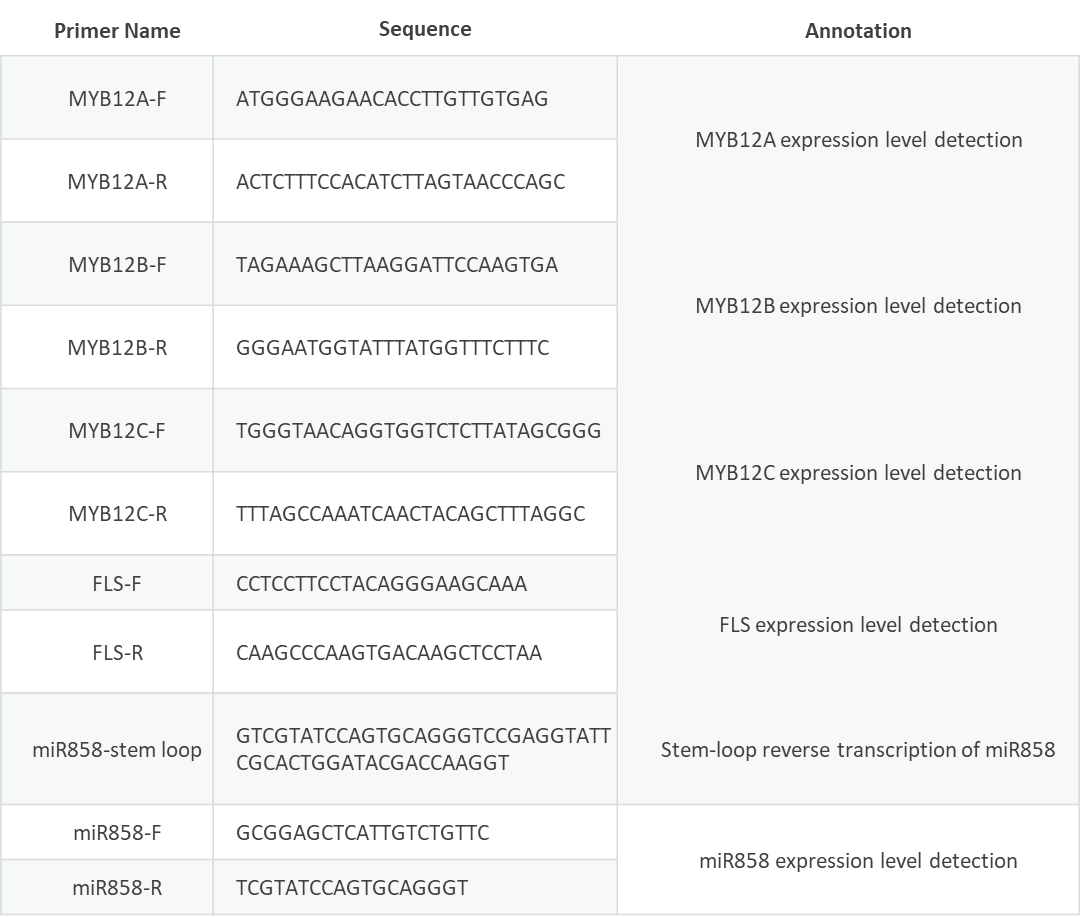
**

**Table S4.** Primers used in this study. Additional primers described in Payyavula et al., 2015.
